# Lifetime body mass index and grip strength at age 46 years: the 1970 British Cohort Study

**DOI:** 10.1002/jcsm.12992

**Published:** 2022-05-19

**Authors:** Rachel Cooper, David Tomlinson, Mark Hamer, Snehal M. Pinto Pereira

**Affiliations:** ^1^ Department of Sport and Exercise Sciences, Musculoskeletal Science and Sports Medicine Research Centre Manchester Metropolitan University Manchester UK; ^2^ Institute of Sport, Exercise and Health, UCL Division of Surgery and Interventional Science University College London London UK

**Keywords:** Grip strength, Obesity, Sarcopenia, Sarcopenic obesity, Life course, Epidemiology

## Abstract

**Background:**

Ongoing rises in obesity prevalence have prompted growing concerns about potential increases in the burden of age‐related musculoskeletal conditions including sarcopenia and sarcopenic obesity. This is of particular concern for future generations of older adults who have lived more of their lives in an obesogenic environment than current generations of older adults. We aimed to study longitudinal associations between body mass index (BMI) and grip strength in midlife using data from a large population‐based sample, the 1970 British Cohort Study (BCS70).

**Methods:**

BCS70 participants with valid measures of maximum grip strength at age 46 years were included in analyses [3671 males (49%) and 3876 females (51%)]. Using sex‐specific linear regression models, we examined associations of (i) BMI at ages 10, 16, 30, and 46 years; (ii) body fat percentage (BF%) and waist–hip ratio at age 46 years; (iii) BMI gains between 10–16, 16–30, and 30–46; and (iv) age at onset of obesity, with grip strength.

**Results:**

At age 46 years, mean (standard deviation) grip strength was 48.10 kg (8.98) in males and 29.61 kg (5.81) in females. Higher BMI at all ages was associated with stronger grip, and the scale of associations was greater in males than females from age 16 onwards (*P*_sex interactions_ < 0.01). For example, in fully adjusted models, a 1 standard deviation increase in BMI at age 16 was associated with mean differences in grip strength at age 46 years of 1.41 kg (95% confidence interval: 1.07, 1.75) in males and 0.72 kg (0.53, 0.91) in females. Higher BF% at age 46 was also associated with stronger grip in both sexes. Greater gains in BMI between ages 10 and 16 were associated with stronger grip in both sexes, but subsequent gains in BMI were only associated with stronger grip in males. Associations of greater length of exposure to obesity and stronger grip were also more consistent among males than females. For example, in fully adjusted models, mean grip strength at age 46 years of males and females who had been obese since age 10 or 16 years was 4.39 kg (1.85, 6.93) and 1.25 kg (−0.18, 2.69) higher than males and females who had never been obese, respectively.

**Conclusions:**

Higher BMI from childhood onwards is associated with stronger grip at age 46 years. This suggests that, at this age, anabolic effects of fat on muscle are outweighing the catabolic effects thought to lead to the manifestation of sarcopenic obesity later in life, especially among men. Midlife may be an optimal time to intervene to prevent sarcopenic obesity.

## Introduction

Age‐related musculoskeletal conditions are leading contributors to the global burden of disease and disability,^1^, ^2^ and evidence suggests that their burden has been increasing in recent decades.^2^ The assignment of an ICD‐10 code to sarcopenia in 2016^3^ reflects growing awareness of the major personal and societal impacts of this specific age‐related musculoskeletal condition^4^ and the important contributions of lower levels of muscle mass and function to the global disease and disability burden.^5^

Muscle weakness, often indicated by low grip strength, is an important indicator of poor muscle function and one of the key criteria for sarcopenia.^5^ A recent study of secular trends in grip strength in older adults in England noted a slight decline in mean grip strength in more recently born cohorts.^6^ This finding is consistent with evidence of increasing levels of mild disability in more recently born cohorts of older adults in England,^7^ as lower grip strength has been shown to be associated with increased subsequent risk of mobility disability.^8^, ^9^ One proposed explanation for the adverse secular trends in grip strength and mild disability is the increasing prevalence of overweight and obesity, with the need for further research to elucidate this highlighted.^6^, ^7^ If obesity does explain the lower mean levels of grip strength in more recently born cohorts of older adults, the implications for the musculoskeletal health and function of future generations of older adults would be very concerning and require urgent action. This is because more recently born generations have lived more of their lives in an obesogenic environment and so are more likely to have become overweight or obese at younger ages than current generations of older adults.^10^, ^11^ Added to which are concerns that this could have been exacerbated since March 2020 by the widespread impacts of strategies taken in many countries to suppress community transmission of COVID‐19 on sedentariness and obesity and the resultant deconditioning of muscle.^12^, ^13^

It is unclear if the rising prevalence of obesity explains the worrying secular trends in grip strength in some countries. There is also a lack of evidence on the key life stages that are critical with respect to opportunities to mitigate any risks associated with obesity. This is because findings from existing studies of associations between lifetime obesity and grip strength are equivocal.^14^, ^15^, ^16^, ^17^ This may be because there are plausible reasons to expect the scale and direction of associations between obesity and grip strength to change with age and length of exposure to obesity. For example, evidence from cross‐sectional physiological studies suggests that fat mass has both anabolic and catabolic effects on muscle.^18^ Initially higher adiposity may promote muscle growth and function as a result of greater loading, especially among men and at younger ages. However, with increased length of exposure to adiposity, these compensatory mechanisms are expected to become less effective, and the catabolic effects of chronic low‐grade inflammation and fat infiltration of muscle manifest.^18^ To further our understanding of these associations and their implications for the musculoskeletal health of future generations of older adults, studies are required that examine longitudinal associations of adiposity and grip strength in more recently born cohorts, who have had greater exposure to overweight and obesity from younger ages. Moreover, as most existing studies have focused on grip strength in older adults, it would also be valuable to examine associations at younger ages before the onset of age‐related declines in grip strength, given this may provide additional insights on opportunities for primary prevention of sarcopenia and sarcopenic obesity.

To address the need for large population‐based studies of longitudinal associations between adiposity and grip strength, we utilized data from the 1970 British Cohort Study (BCS70) to investigate associations of (i) body mass index (BMI) in childhood, adolescence, and early adulthood; (ii) BMI gain during different life stages; and (iii) age at onset of obesity, with grip strength at age 46 years. To assess whether any associations of BMI and grip strength were driven by fat mass and the importance of body fat distribution, we also examined cross‐sectional associations of body fat percentage (BF%) and waist–hip ratio (WHR) with grip strength.

## Subjects and methods

**Figure 1:**
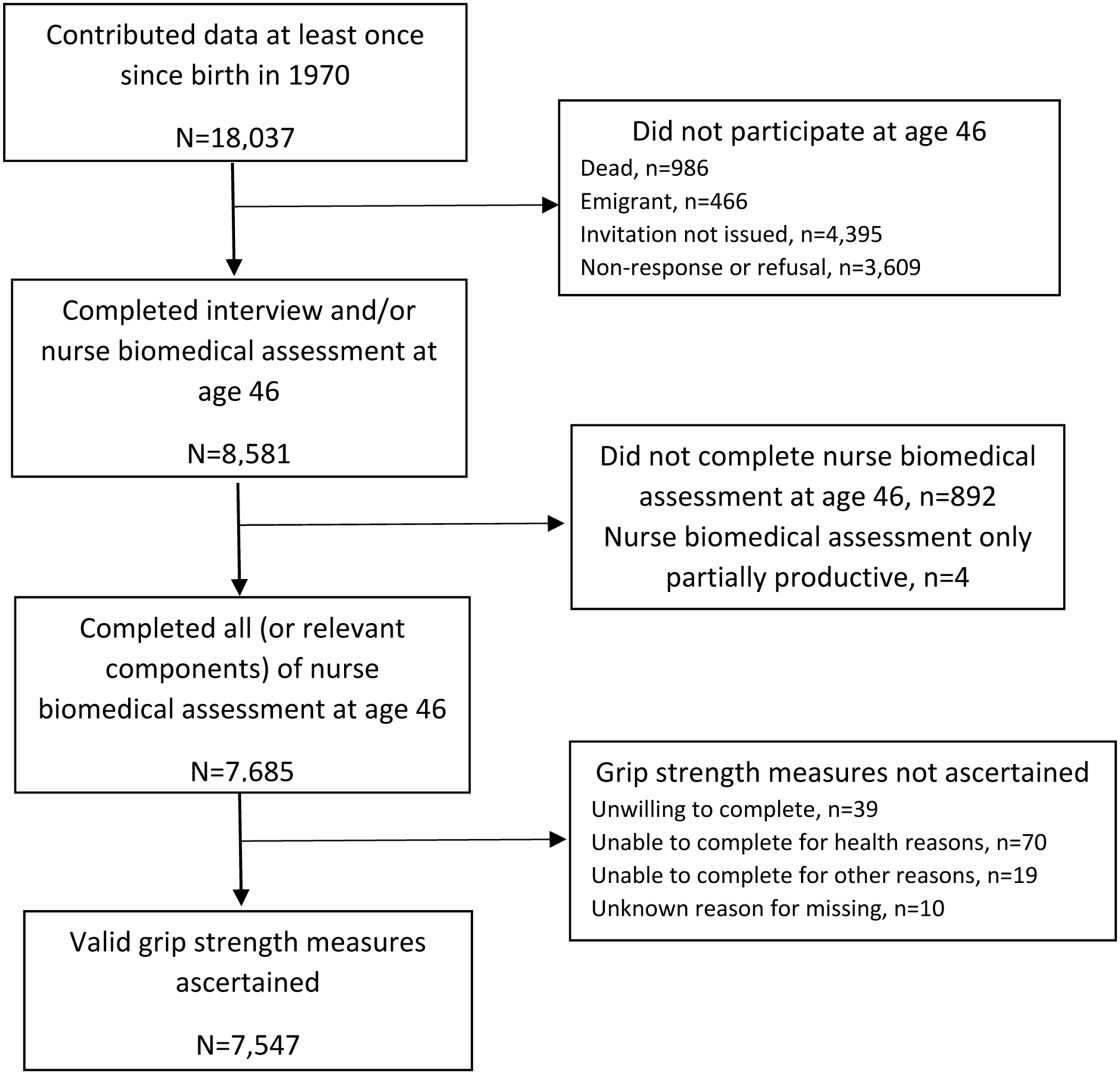
Flow diagram of participation in the 1970 British Cohort Study.

The BCS70 comprises males and females born in England, Scotland, and Wales during a single week in 1970 plus people born in other countries in the same week who moved to Great Britain during childhood.^19^ Study participants who were recruited at birth (or in the case of immigrants, during one of three main data collections in childhood) have been followed up and assessed regularly across life. During an assessment in 2016–18, when participants were aged 46 years, a home visit was conducted involving 50 min of interviews (both face‐to‐face computer‐assisted personal interview and computer‐assisted self‐completion interview) and a 50 min nurse‐led biomedical assessment.^20^ Of the 18 037 males and females documented to have participated in one or more of the main data collections across life,^21^ 8581 completed at least one component of the assessment at age 46.^22^ Of these participants, 7685 completed a nurse biomedical assessment and 7547 (3671 males and 3876 females) had valid grip strength measures (details in *Figure* 1). Participants provided informed consent and the assessment at age 46 years received full ethical approval from NRES Committee South East Coast—Brighton and Sussex (Ref. 15/LO/1446).

### Body mass index and other indicators of body composition

Body mass index (kg/m^2^) was calculated using measured heights and weights at ages 10, 16, and 46 years and self‐reported height and weight at age 30 years. As heights and weights were measured over a range of ages at each assessment (e.g. ages 9.7–11.7 at the 10 year follow‐up), BMI was centred at 10, 16, 30, and 46 years, respectively, by using predictions from linear regression models that assumed a linear age trend over short periods. A 1 kg/m^2^ unit of BMI has different implications in childhood and adulthood. To ensure comparability of models, BMI at each age was sex standardized to a mean of 0 and standard deviation (SD) of 1 [in all cases, this was calculated as (*x* − mean)/SD where *x* is the individual's BMI and mean and SD represent the sample mean and SD of BMI for all males or females at the specified age]. As a result, our unit of analysis for BMI at all ages was 1SD.

To estimate duration of exposure to obesity, BMI at each age was categorized as not obese vs. obese using recommended age‐specific and sex‐specific cut‐points at ages 10 and 16^23^ and standard cut‐points for adulthood at ages 30 and 46 (i.e. <30 vs. ≥30 kg/m^2^).^24^ A variable indicating the youngest age at which each participant was first classified as obese was then derived, with never obese as the reference category.

At age 46 years, BF% was measured via bio‐impedance using Tanita BF‐522W scales. The circumferences of the waist (midway between the iliac crest and the costal margin) and hips (widest circumference over the buttocks and below the iliac crest), to the nearest millimetre, were measured by nurses following standardized protocols. Two valid measures of both waist and hip circumferences were recorded and the averages used to estimate WHR. Participants were excluded from these measurements if they were pregnant or unable to stand. In addition, participants weighing >130 kg and those with a pacemaker or internal defibrillator were excluded from bio‐impedance measures, and those who had a colostomy/ileostomy were excluded from waist and hip measurements. To facilitate comparisons across all models, similar to BMI, BF% and WHR were sex standardized.

### Grip strength

During the biomedical assessment at age 46 years, nurses measured grip strength following a standardized protocol. Participants were instructed to hold a Smedley spring‐gauge hand‐held dynamometer and squeeze its handle as hard as they could for 2 s with the value achieved in kg recorded by the nurse before the device was reset. Participants were asked to stand without arm support but were allowed to conduct the test with arm support and seated if required. The test was performed up to six times, three times in each hand, alternating between hands. The maximum grip strength (kg) achieved was used in analyses. Participants were not asked to complete the grip strength test if they reported swelling or inflammation, severe pain, or a recent injury to their hands or had surgery on their hands in the last 6 months with these and other reasons for being unable to complete the assessment recorded by the nurse (*Figure* 1).

### Covariates

Covariates were selected a priori based on previous literature. As height and grip strength are strongly correlated^25^ and grip strength is often normalized for height,^26^ we first chose to adjust for height, measured by nurses at age 46 years. Other covariates were socio‐economic position (SEP) in childhood [indicated by father's occupation at birth (or at age 10 if missing) categorized according to the Registrar General's Social Classification (RGSC)]; physical activity at age 10 years (based on maternal reports of their child's level of participation in sports); SEP in adulthood [indicated by own occupational class at age 46 years (using the same categories as for father's occupation after back‐coding National Statistics Socio‐economic Classifications of occupations to RGSC^27^) and highest educational level attained by age 46 years]; and physical activity at age 46 years (based on self‐reports of how many days per week participants reported exercising for ≥30 min causing them to experience an elevated heart rate and sweating). Categorizations of each covariate are presented in *Table* 1.

**Table 1:** Characteristics of the 1970 British Cohort Study participants included in analyses {sample restricted to those with valid measures of grip strength at age 46 years [maximum *N* = 7547^a^ (3671 males and 3876 females)]}

	Mean (SD) or *N* (%)	
	Males	Females
Grip strength (kg)	48.10 (8.98)	29.61 (5.81)
BMI (kg/m^2^) at age		
10 years	16.70 (1.90)	16.89 (2.19)
16 years	20.69 (2.96)	21.08 (3.15)
30 years	25.52 (3.84)	24.10 (4.78)
46 years	28.90 (5.11)	28.38 (6.54)
Body fat %, 46 years	23.42 (8.30)	36.93 (7.44)
Waist–hip ratio, 46 years	0.94 (0.07)	0.83 (0.07)
Height (m), 46 years	1.78 (0.08)	1.64 (0.07)
Father's occupational class at birth^b^		
I/II	777 (22.2)	777 (21.0)
IIINM	502 (14.4)	523 (14.2)
IIIM	1615 (46.2)	1707 (46.2)
IV/V	603 (17.2)	688 (18.6)
Physical activity at age 10 years		
Never/hardly ever	158 (5.0)	359 (10.7)
Sometimes	915 (28.7)	1632 (48.5)
Often	2114 (66.3)	1376 (40.9)
Highest educational level by age 46 years^c^		
No formal qualifications or <O‐levels	1356 (37.6)	1185 (30.9)
O‐levels or GCSEs	870 (24.1)	997 (26.0)
A‐levels	201 (5.6)	228 (6.0)
University degree or higher	1182 (32.8)	1423 (37.1)
Own occupational class at age 46 years^b^		
I/II	1606 (47.8)	1406 (44.8)
IIINM	468 (13.9)	986 (31.4)
IIIM	956 (28.4)	324 (10.3)
IV/V/long‐term unemployed	332 (9.9)	420 (13.4)
Physical activity (days/week) at age 46 years		
0	717 (19.8)	1014 (26.5)
1	357 (9.8)	359 (9.4)
2	447 (12.3)	499 (13.1)
3	496 (13.7)	591 (15.5)
4/5	817 (22.5)	682 (17.9)
6/7	793 (21.9)	676 (17.7)

BMI, body mass index; SD, standard deviation.

^a^ *N*s presented in the table vary due to missing data.

^b^ Registrar General's Social Classification: I/II = Professional or Managerial and Technical; IIINM = Skilled Non‐manual; IIIM = Skilled Manual; IV/V = Semi‐skilled or Unskilled.

^c^ O levels and GCSES = standard qualifications obtained within the British school system at age 16; A levels = the highest qualification that can be obtained within the British school system (usually at age 18).

### Statistical analyses

We decided a priori to stratify analyses by sex as previous studies have reported sex differences in BMI–grip strength associations.^17^ We formally assessed whether there were differences in association by sex with tests of sex interaction.

We ran a series of linear regression models to test associations of (1) BMI at each age; (2) BF% and WHR at age 46; and (3) age at onset of obesity, with grip strength at age 46 years. In initial models, we included quadratic terms for BMI, BF%, and WHR (as appropriate) to assess deviations from linearity. Models were adjusted for covariates in stages with height at 46 added first, followed by childhood covariates (father's occupational class and physical activity at age 10), then SEP and physical activity at age 46.

To investigate associations between BMI gain during different life stages and grip strength, we calculated the change in BMI for the periods 10–16, 16–30, and 30–46 years conditional on earlier BMI by regressing each BMI measure on the earlier measure(s) for each sex and saving the residuals. These residuals can be interpreted as the change in BMI above or below that expected given earlier BMI. The residuals were standardized (mean = 0 and SD = 1) to allow a comparison of the relative associations of changes in BMI in different periods with grip strength. Linear regression models were fitted that included all three standardized residuals with grip strength as the outcome. We formally assessed whether the coefficients for 10–16 and 30–46 differed from the coefficient for 16–30. Models were run unadjusted and with adjustment for all covariates.

To minimize bias, missing values of the main explanatory factors and covariates in the sample with valid data on grip strength (*n* = 7547) were imputed [missing data ranged from 0.01% (adult height) to 39% (for height at 16 years)] using multiple imputation chained equations. All analyses were run across 20 imputed datasets and estimates were combined using Rubin's rules.^28^ Imputed results from unadjusted models were broadly similar to those obtained using observed values (Supporting Information, *Table* S1); only results from imputed analyses are presented as follows.

### Sensitivity analyses

A series of sensitivity analyses were undertaken to check that the main findings were not (1) influenced by variation between participants in their positioning during grip strength assessment; (2) driven by the inclusion of people with disability; or (3) conversely, influenced by the exclusion of people unable to complete the grip strength assessment for health reasons. To do this, we reran unadjusted and fully adjusted models of associations of BMI at each age and BF% and WHR at age 46 with grip strength with (1) inclusion of only those participants who completed the grip strength test standing without arm support (*N* = 6890); (2) exclusion of participants classified as being severely hampered according to the European Statistics of Income and Living Conditions (EU‐SILC) classification^29^ disability definition (*n* = 452) or with missing disability information (*n* = 3); and (3) inclusion of 70 additional participants unable to complete the grip strength tests for health reasons by allocating them grip strength values equivalent to the mean of the bottom sex‐specific fifth.

## Results

Characteristics of the 7547 males and females from BCS70 with valid grip strength measures at age 46 years are presented in *Table* 1. As expected, males had higher mean grip strength than females and mean BMI increased between ages 10 and 46 years in both sexes. Females had higher BF% at age 46 than males and in contrast males had higher WHR.

**Figure 2:**
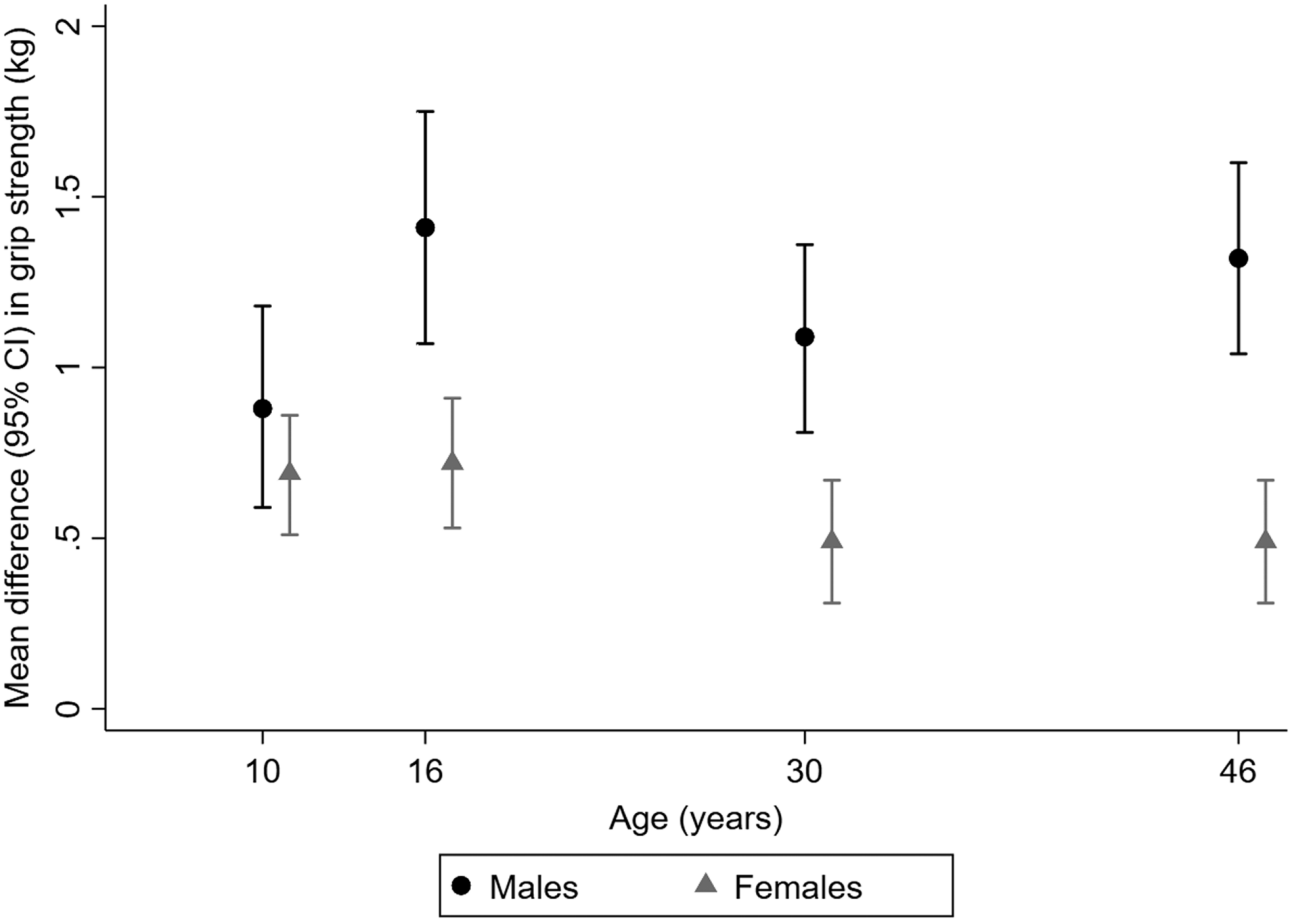
Differences in mean grip strength at age 46 years per 1 standard deviation increases in body mass index at ages 10 to 46 years in males and females (*N* = 7547). Estimates from models adjusted for father's occupational class at birth, physical activity at age 10 years, educational level attained, own occupational class, height, and physical activity at age 46 years. For more details, see *Table* 2. Results are combined from analyses run across 20 imputed datasets. CI, confidence interval.

In both males and females, higher BMI at all ages was associated with stronger grip and these associations were maintained after adjustment for covariates (*Table* 2 and *Figure* 2). Associations between BMI and grip strength were stronger in males than females from age 16 years onwards (*P*_sex interaction_ < 0.001). For example, in fully adjusted models, a 1SD increase in BMI at age 16 was associated with mean differences in grip strength at age 46 years of 1.41 kg (95% confidence interval: 1.07, 1.75) in males and 0.72 kg (0.53, 0.91) in females. Although some evidence of deviations from linearity was suggested when quadratic terms were included in models of BMI (*Table* S2), when plots of the associations between BMI at each age and grip strength at age 46 years were examined (*Figure* S1), these suggested that any deviations from linearity were minor and due to extreme values. In addition, when BMI was modelled in standard categories (*Table* S3), conclusions remained the same.

**Table 2:** Differences in mean grip strength at age 46 years per 1 standard deviation increase in BMI at ages 10 to 46 years and in BF% and WHR at age 46 years (*N* = 7547)

	Model	Differences in mean grip strength (kg) (95% CI)	
		Males (*N* = 3671)	Females (*N* = 3876)
BMI at 10 years	1	0.79 (0.49, 1.09)	0.62 (0.43, 0.81)
	2	0.86 (0.57, 1.16)	0.66 (0.48, 0.84)
	3	0.89 (0.59, 1.19)	0.68 (0.51, 0.86)
	4	0.88 (0.59, 1.18)	0.69 (0.51, 0.86)
BMI at 16 years	1	1.36 (1.01, 1.70)	0.60 (0.40, 0.80)
	2	1.46 (1.12, 1.81)	0.70 (0.51, 0.90)
	3	1.45 (1.10, 1.79)	0.70 (0.51, 0.89)
	4	1.41 (1.07, 1.75)	0.72 (0.53, 0.91)
BMI at 30 years	1	1.05 (0.77, 1.33)	0.20 (0.02, 0.39)
	2	1.12 (0.85, 1.40)	0.38 (0.20, 0.56)
	3	1.10 (0.83, 1.38)	0.41 (0.23, 0.59)
	4	1.09 (0.81, 1.36)	0.49 (0.31, 0.67)
BMI at 46 years	1	1.26 (0.97, 1.55)	0.19 (0.01, 0.38)
	2	1.27 (0.99, 1.55)	0.33 (0.15, 0.51)
	3	1.26 (0.98, 1.54)	0.38 (0.20, 0.56)
	4	1.32 (1.04, 1.60)	0.49 (0.31, 0.67)
BF% at 46 years	1	0.36 (0.08, 0.65)	0.26 (0.08, 0.43)
	2	0.37 (0.09, 0.65)	0.12 (−0.05, 0.29)
	3	0.35 (0.07, 0.63)	0.17 (−0.01, 0.34)
	4	0.39 (0.11, 0.67)	0.28 (0.10, 0.45)
WHR at 46 years	1	−0.13 (−0.42, 0.16)	−0.17 (−0.35, 0.01)
	2	−0.10 (−0.39, 0.18)	−0.05 (−0.22, 0.13)
	3	−0.10 (−0.39, 0.19)	−0.01 (−0.19, 0.16)
	4	0.00 (−0.30, 0.29)	0.06 (−0.12, 0.23)

BF%, body fat percentage; BMI, body mass index; CI, confidence interval; WHR, waist–hip ratio.

Model adjustments: 1: unadjusted (*P*‐values from formal tests of sex interaction, *P* = 0.44 for BMI at age 10, *P* < 0.001 for BMI at ages 16, 30, and 46 years, *P* = 0.53 for BF% and *P* = 0.83 for WHR); 2: adjusted for height at 46 years; 3: Model 2 + father's occupational class at birth and physical activity at age 10 years; 4: Model 3 + educational level attained, own occupational class, and physical activity at age 46 years. Results are combined from analyses run across 20 imputed datasets.

At age 46 years, higher BF% was also associated with stronger grip in both sexes, but associations were weaker than those observed for BMI at 46 especially among males (*Table* 2). For example, for males, in fully adjusted models, 1SD increases in BF% and BMI at 46 were associated with mean differences in grip strength of 0.39 kg (0.11, 0.67) and 1.32 kg (1.04, 1.60), respectively. There was no clear evidence of association between WHR and grip strength at age 46 in either sex.

**Figure 3:**
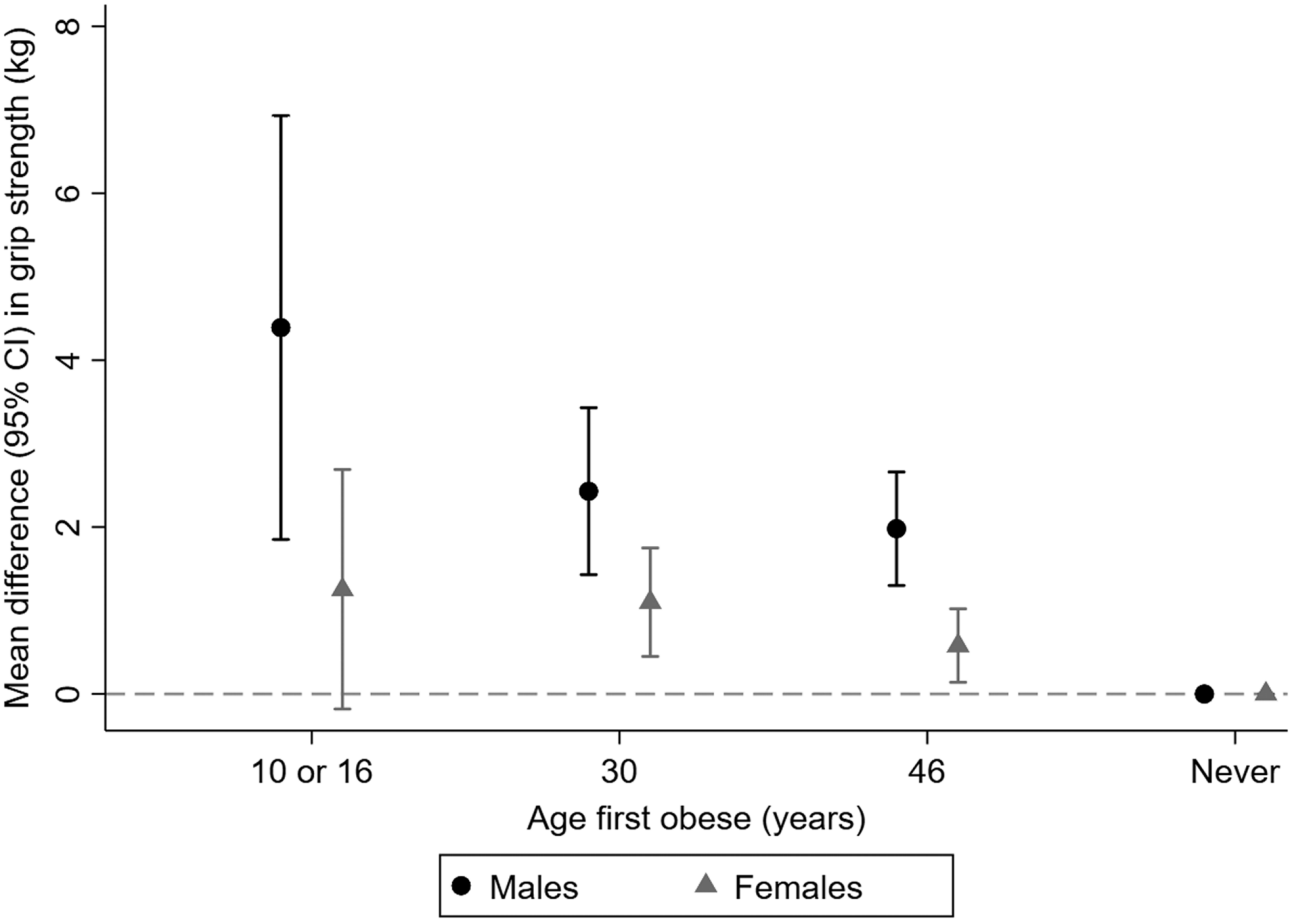
Differences in mean grip strength at age 46 years by age first obese in males and females (*N* = 7547). Estimates from models adjusted for father's occupational class at birth, physical activity at age 10 years, educational level attained, own occupational class, height, and physical activity at age 46 years. For more details, see *Table* 4. Results are combined from analyses run across 20 imputed datasets. CI, confidence interval.

Greater gains in BMI between ages 10 and 16 years were associated with stronger grip in both sexes (*Table* 3). Greater gains in BMI in later age periods were also associated with stronger grip in males though associations were weaker. Among females, there was no clear evidence of association between BMI gains between ages 16 and 30 or 30 and 46 years and grip strength. Consistent with these findings, there was a clear pattern of association between greater length of exposure to obesity and stronger grip among males but not among females (*Table* 4 and *Figure* 3). For example, males who were first classified as obese at 10 or 16 years had mean grip strength 4.39 kg (95% confidence interval: 1.85, 6.93) higher than males who had never been obese in fully adjusted models, but the equivalent estimate in females was 1.25 kg (−0.18, 2.69).

**Table 3:** Differences in mean grip strength at age 46 years per 1 standard deviation increase in BMI over specified age intervals (conditional on prior BMI) (*N* = 7547)

Interval of BMI change	Differences in mean grip strength (kg) (95% CI)			
	Males (*N* = 3671)	*P*‐value^a^	Females (*N* = 3876)	*P*‐value^a^
Unadjusted				
10–16 years	1.02 (0.65, 1.40)	0.07	0.27 (0.06, 0.49)	<0.01
16–30 years	0.54 (0.23, 0.85)	—	−0.20 (−0.40, −0.01)	—
30–46 years	0.53 (0.24, 0.83)	0.98	−0.03 (−0.22, 0.16)	0.21
Fully adjusted^b^				
10–16 years	1.02 (0.65, 1.40)	0.07	0.37 (0.17, 0.57)	0.03
16–30 years	0.55 (0.25, 0.85)	—	0.06 (−0.12, 0.25)	—
30–46 years	0.55 (0.25, 0.84)	0.99	0.10 (−0.08, 0.28)	0.80

BMI, body mass index; CI, confidence interval.

Results are combined from analyses run across 20 imputed datasets.

^a^ *P*‐value from formal test of difference between coefficient and 16–30 years coefficient.

^b^ Model adjusted for height at 46 years, father's occupational class at birth and physical activity at age 10 years, educational level attained, own occupational class, and physical activity at age 46 years (for brevity, results from Models 2 and 3, as per *Table* 2, are not presented).

**Table 4:** Differences in mean grip strength at age 46 years by age first obese (*N* = 7547)

		Differences in mean grip strength (kg) (95% CI)			
		Males (*N* = 3671)		Females (*N* = 3876)	
		%^a^		%^a^	
Unadjusted					
Age first obese	Never	63.5	Reference	65.9	Reference
	46 years	24.5	1.95 (1.26, 2.65)	23.0	0.08 (−0.38, 0.53)
	30 years	10.4	2.34 (1.31, 3.36)	9.2	0.25 (−0.43, 0.93)
	10 or 16 years	1.6	3.44 (0.91, 5.98)	1.9	0.72 (−0.84, 2.27)
Fully adjusted^b^					
Age first obese	Never		Reference		Reference
	46 years		1.98 (1.30, 2.66)		0.58 (0.14, 1.02)
	30 years		2.43 (1.43, 3.43)		1.10 (0.45, 1.75)
	10 or 16 years		4.39 (1.85, 6.93)		1.25 (−0.18, 2.69)

CI, confidence interval.

Results are combined from analyses run across 20 imputed datasets.

^a^ Averaged over 20 datasets.

^b^ Model adjusted for height at 46 years, father's occupational class at birth and physical activity at age 10 years, educational level attained, own occupational class, and physical activity at age 46 years (for brevity, results from Models 2 and 3, as per *Tabl*e 2, are not presented).

Sensitivity analyses confirmed that our results were not influenced by (1) participant's position during grip strength testing (*Table* S4); (2) including participants classified as severely hampered (*Table* S5); or (3) excluding people unable to complete the grip strength assessments for health reasons (*Table* S6).

## Discussion

In a population‐based study of over 7000 males and females followed from birth for almost five decades, we found robust evidence that higher BMI from childhood onwards is associated with stronger grip at age 46 years in both males and females. Notably, there were clear sex differences—the scale of associations from age 16 years was greater, and there was more consistent evidence that associations were cumulative in males than females. While cross‐sectional associations between higher BF% and stronger grip were also observed, these were weaker than associations with BMI especially among males. There was no evidence of association between WHR and grip strength in either sex.

Our work is novel and adds important new insights to the existing evidence base by exploring longitudinal associations in a more recently born cohort with a younger age at assessment of grip strength than previous studies. Our finding of positive relationships between BMI from childhood onwards and grip strength is consistent with and builds on evidence of cross‐sectional associations between higher BMI and stronger grip in adults aged 48–92 years in the European Prospective Investigation into Cancer‐Norfolk study^16^ and in male participants aged 50 to 90+ from 8 UK cohort studies that contributed to the Healthy Ageing across the Life Course (HALCyon) research programme.^17^ In HALCyon, similar associations were not observed in women, consistent with our finding of weaker associations in females than males. Our findings are in contrast to one of the only other large population‐based studies that utilized longitudinal data and explored cumulative associations: in Finnish adults aged 55 years and over, greater length of exposure to obesity (defined using BMI) across adulthood was associated with weaker grip strength.^14^ The use of retrospective self‐reports of weight history and the older age of the Finnish sample may explain our contrasting findings. However, this inconsistency also serves to highlight the complexity of associations and the need to carefully consider (i) how associations may change with age, period, cohort, and place and (ii) the extent to which the patterns of association observed are dependent on the measures of adiposity and strength utilized.

One potential explanation of associations between higher BMI from childhood onwards and stronger grip at age 46 years is that fat mass acts as a mechanical load that elicits anabolic responses promoting muscle growth and function, especially earlier in life.^18^ As this anabolic response is usually greater in males than females due to higher circulating levels of testosterone, this may contribute to observed sex differences in associations. However, alternative explanations also need to be considered. As BMI does not distinguish between lean and fat mass, its reliability as an indicator of adiposity may vary by sex. In BCS70 at age 46 years, the correlation between BMI and BF% (a more direct measure of fat mass) was 0.66 in males vs. 0.82 in females. That higher BMI at any one age and gains in BMI over time are more likely to reflect higher levels of muscle mass and greater muscle mass accrual, respectively, in males than females suggests that BMI in males might be less representative of fat mass than in females. This could explain why we found stronger effects and more consistent evidence of cumulative associations in males. It could also explain the greater discrepancy in the scale of cross‐sectional associations between BMI and BF% with grip strength observed in males than females and the finding of no sex difference in associations between BF% and grip strength.

Despite these recognized limitations, we selected to use BMI as our primary indicator of adiposity. This was because it had been measured prospectively at multiple ages allowing us to add a life course perspective to the existing evidence base, which is an important and novel aspect of our work. Our longitudinal analyses of BMI were complemented by cross‐sectional analyses of BF% and WHR, which unfortunately had not been measured at earlier ages. Insights provided by our findings on BF% suggest that further work to elucidate associations using longitudinal data on more accurate measures of body composition that clearly distinguish between fat and lean mass would be valuable. However, there is currently a dearth of studies that have such data coupled with muscle function assessments in adulthood. Another key strength of our study is the use of a large, population‐based sample who were nationally representative at birth and have been followed prospectively since. As with all long‐term studies, we acknowledge that due to losses to follow‐up and non‐response, bias may have been introduced limiting the generalizability of our findings somewhat—BCS70 participants included in our analyses were more likely to be female and normal weight in childhood and adulthood and have a higher lifetime SEP and better self‐rated health at age 46 than those not included. However, we maximized our analytic sample and minimized potential bias due to missing data by using multiple imputation. Another strength of our work was the availability of prospectively ascertained data on a number of potentially important covariates and the use of a series of sensitivity analyses to assess the robustness of our findings. Residual confounding does however remain a possible explanation of our findings.

Our results need to be interpreted with some caution because for any given bodily movement (especially those that are weight bearing), people with greater body mass will require more muscle strength than people with lower body mass. However, by studying absolute grip strength, we have used the recommended measure for detecting low muscle strength when defining sarcopenia.^30^ This ensures comparability with other studies, and as strength was assessed before the onset of major age‐related declines in our study, the results have implications for the primary prevention of sarcopenia and sarcopenic obesity. That higher BMI from childhood onwards was associated with stronger grip at age 46 years and, in cross‐sectional analyses, higher BF% was also associated with stronger grip but WHR was not suggests that catabolic effects of fat on muscle function, related to chronic inflammation, insulin resistance, and hormone dysregulation, that are thought to underlie the development of sarcopenic obesity by later life are not manifest by midlife (i.e. age 46).^18^, ^31^, ^32^ In the MRC National Survey of Health and Development, another British cohort born in 1946, there was evidence of associations between higher BMI across adulthood and lower muscle quality by ages 60–64 years.^15^ This demonstrates the importance of monitoring a range of different parameters of muscle. More importantly, it highlights that midlife (i.e. between the mid‐40s and early 60s) may represent an optimal time to intervene to prevent sarcopenic obesity.

In identifying other implications of our work, it is important to consider recent findings from UK Biobank showing that obesity was associated with greater likelihood of having stable high grip strength but also with greater risk of grip strength decline over 9 years of follow‐up.^33^ This, taken together with our findings, would suggest that some people who have had greater lifetime exposure to obesity may require more support than others to maintain their strength in later life. Our findings also suggest the need to ensure that weight loss programmes for overweight and obese adults in midlife, which are widely promoted to mitigate the many adverse health effects of obesity, target fat mass specifically and that care is taken to ensure they do not detrimentally impact on muscle.

There are currently fewer than 100 BCS70 participants with low muscle strength (as indicated by a grip strength of <27 kg in males and <16 kg in females)^30^ because of their relatively young age. However, as the cohort ages, future follow‐ups should provide interesting opportunities for further detailed investigations of lifetime obesity in relation to grip strength focusing on different patterns of age‐related change in grip strength and the role of potential modifiers and mediators in the development of sarcopenia and sarcopenic obesity.

In conclusion, our study of British adults in midlife has provided important new insights on the complex life course associations between obesity and grip strength. That higher BMI from childhood onwards is associated with stronger grip at age 46 years suggests that, at this age, anabolic effects of fat on muscle are outweighing the catabolic effects thought to lead to the manifestation of sarcopenic obesity later in life, especially among males. Midlife may therefore represent an optimal time to intervene to prevent sarcopenic obesity in old age.

## Funding

The biomedical assessment of the 1970 British Cohort Study at age 46 years was funded by a joint award from the Economic and Social Research Council and Medical Research Council (RES‐579‐47‐0001). S.M.P.P. is supported by a UK Medical Research Council Career Development Award (Ref. MR/P020372/1).

The funders had no role in the study design, in the collection, analysis, and interpretation of data, in writing of the report, or in the decision to submit the paper for publication.

## Conflicts of interest

M.H. has received an unrestricted grant from PAL Technologies, Scotland, UK. R.C., D.T., and S.M.P.P. have no competing interests to disclose.

## Supporting information


**Table S1.** Unadjusted differences in mean grip strength at age 46y per 1 standard deviation increase in body mass index (BMI) at ages 10 to 46y and in body fat percentage (BF%) and waist hip ratio (WHR) at age 46y on observed data
**Table S2.** Unadjusted differences in mean grip strength at age 46y per 1 standard deviation increase in BMI at ages 10 to 46y and in BF% and WHR at age 46y with the inclusion of quadratic terms (*N* = 7,547)
**Table S3.** Unadjusted differences in mean grip strength at age 46y by standard categories of BMI at ages 10 to 46 years (*N* = 7,547)
**Table S4.** Unadjusted (model 1) and fully‐adjusted (model 4) differences in mean grip strength at age 46y per 1 standard deviation increase in BMI at ages 10 to 46y and in BF% and WHR at age 46y among the sample who completed the grip strength assessment standing unsupported (*N* = 6,890)
**Table S5.** Unadjusted (model 1) and fully‐adjusted (model 4) differences in mean grip strength at age 46y per 1 standard deviation increase in BMI at ages 10 to 46y and in BF% and WHR at age 46y excluding those participants classified as severely hampered according to the European Statistics of Income and Living Conditions (EU‐SILC) classification disability definition or with missing disability data (*N* = 7.092)
**Table S6.** Unadjusted (model 1) and fully‐adjusted (model 4) differences in mean grip strength at age 46y per 1 standard deviation increase in BMI at ages 10 to 46y and in BF% and WHR at age 46y with inclusion of those participants unable to complete the grip strength assessments for health reasons* (*N* = 7,617)
**Figure S1.** Scatter plots and locally weighted regressions of BMI (kg/m^2^) vs grip strength (kg) for males at 16y and 46y
