# Supplementary material for: Lifetime body mass index and grip strength at age 46 years: the 1970 British Cohort Study
Source: J Cachexia Sarcopenia Muscle. 2022 May 19;13(4):1995–2004. doi: 10.1002/jcsm.12992 (PMC9397548; doi:10.1002/jcsm.12992)
Supplement: Supplementary file 1 — Table S1. Unadjusted differences in mean grip strength at age 46y per 1 standard deviation increase in body mass index (BMI) at ages 10 to 46y and in body fat percentage (BF%) and waist hip ratio (WHR) at age 46y on observed data Table S2. Unadjusted differences in mean grip strength at age 46y per 1 standard deviation increase in BMI at ages 10 to 46y and in BF% and WHR at age 46y with the inclusion of quadratic terms (N = 7,547) Table S3. Unadjusted differences in mean grip strength at age 46y by standard categories of BMI at ages 10 to 46 years (N = 7,547) Table S4. Unadjusted (model 1) and fully‐adjusted (model 4) differences in mean grip strength at age 46y per 1 standard deviation increase in BMI at ages 10 to 46y and in BF% and WHR at age 46y among the sample who completed the grip strength assessment standing unsupported (N = 6,890) Table S5. Unadjusted (model 1) and fully‐adjusted (model 4) differences in mean grip strength at age 46y per 1 standard deviation increase in BMI at ages 10 to 46y and in BF% and WHR at age 46y excluding those participants classified as severely hampered according to the European Statistics of Income and Living Conditions (EU‐SILC) classification disability definition or with missing disability data (N = 7.092) Table S6. Unadjusted (model 1) and fully‐adjusted (model 4) differences in mean grip strength at age 46y per 1 standard deviation increase in BMI at ages 10 to 46y and in BF% and WHR at age 46y with inclusion of those participants unable to complete the grip strength assessments for health reasons* (N = 7,617) Figure S1. Scatter plots and locally weighted regressions of BMI (kg/m2) vs grip strength (kg) for males at 16y and 46y [file JCSM-13-1995-s001.docx]

**Supporting Information for: Lifetime body mass index and grip strength at age 46y: the 1970 British Cohort Study**

Rachel Cooper, David Tomlinson, Mark Hamer, Snehal M Pinto Pereira

Corresponding author: r.cooper@mmu.ac.uk

**Supporting information table S1: Unadjusted differences in mean grip strength at age 46y per 1 standard deviation increase in body mass index (BMI) at ages 10 to 46y and in body fat percentage (BF%) and waist hip ratio (WHR) at age 46y on observed data**

|  |  | **Differences in mean grip strength (kg) (95% CI)** | | | |
| --- | --- | --- | --- | --- | --- |
|  | **N** | **Males** |  | **N** | **Females** |
| BMI at age: |  |  |  |  |  |
| 10y | 2875 | 0.66 (0.34,0.99) |  | 3091 | 0.60 (0.40,0.81) |
| 16y | 2021 | 1.21 (0.83,1.58) |  | 2510 | 0.59 (0.37,0.81) |
| 30y | 3058 | 1.14 (0.82,1.45) |  | 3368 | 0.33 (0.13,0.52) |
| 46y | 3660 | 1.29 (1.00,1.58) |  | 3851 | 0.22 (0.03,0.40) |
| BF% at 46y | 3506 | 0.28 (-0.01,0.58) |  | 3718 | 0.27 (0.08,0.45) |
| WHR at 46y | 3605 | -0.11 (-0.40,0.18) |  | 3793 | -0.21 (-0.39,-0.02) |

**Supporting information table S2: Unadjusted differences in mean grip strength at age 46y per 1 standard deviation increase in BMI at ages 10 to 46y and in BF% and WHR at age 46y with the inclusion of quadratic terms (N=7,547)**

|  |  | **Differences in mean grip strength (kg) (95% CI)** | | |
| --- | --- | --- | --- | --- |
|  |  | **Males (N=3,671)** |  | **Females (N=3,876)** |
| BMI at age: |  |  |  |  |
| 10y | Linear  Quadratic | 1.02 (0.65,1.39)  -0.17 (-0.34,0.00) |  | 0.71 (0.49,0.93)  -0.08 (-0.19,0.03) |
|  |  |  |  |  |
| 16y | Linear  Quadratic | 1.74 (1.35,2.13)  -0.34 (-0.47,-0.20) |  | 0.73 (0.50,0.95)  -0.13 (-0.22,-0.03) |
|  |  |  |  |  |
| 30y | Linear  Quadratic | 1.40 (1.07,1.74)  -0.14 (-0.23,-0.04) |  | 0.31 (0.08,0.53)  -0.04 (-0.08,0.01) |
|  |  |  |  |  |
| 46y | Linear  Quadratic | 1.68 (1.35,2.01)  -0.30 (-0.42,-0.18) |  | 0.43 (0.20,0.65)  -0.17 (-0.27,-0.07) |
|  |  |  |  |  |
| BF% at 46y | Linear  Quadratic | 0.41 (0.11,0.71)  -0.12 (-0.33,0.10) |  | 0.26 (0.08,0.43)  -0.14 (-0.26,-0.02) |
|  |  |  |  |  |
| WHR at 46y | Linear  Quadratic | -0.11 (-0.40,0.18)  -0.20 (-0.39,-0.01) |  | -0.16 (-0.34,0.02)  -0.10 (-0.20,0.00) |

Note: results are combined from analyses run across 20 imputed datasets

**Supporting information table S3: Unadjusted differences in mean grip strength at age 46y by standard categories of BMI at ages 10 to 46 years (N=7,547)**

|  |  | **Differences in mean grip strength (kg) (95% CI)** | | |
| --- | --- | --- | --- | --- |
| Age (years) |  | **Males (N=3,671)** |  | **Females (N=3,876)** |
| 10y | Underweight/Normal  Overweight  Obese | Ref  0.80 (-0.48,2.09)  3.30 (-2.62,9.22) |  | Ref  1.39 (0.72,2.07)  0.28 (-2.43,2.98) |
|  |  |  |  |  |
| 16y | Underweight/Normal  Overweight  Obese | Ref  1.98 (0.66,3.30)  2.72 (0.06,5.38) |  | Ref  0.95 (0.24,1.67)  1.00 (-0.79,2.78) |
|  |  |  |  |  |
| 30y | Underweight/Normal  Overweight  Obese | Ref  1.83 (1.17,2.50)  2.61 (1.63,3.60) |  | Ref  0.40 (-0.08,0.88)  0.43 (-0.21,1.06) |
|  |  |  |  |  |
| 46y | Underweight/Normal  Overweight  Obese | Ref  2.24 (1.48,3.00)  3.56 (2.77,4.36) |  | Ref  0.55 (0.11,1.00)  0.47 (0.03,0.91) |

Note: results are combined from analyses run across 20 imputed datasets

Standard cut-points for overweight and obese in childhood^1^ and adulthood^2^ were applied to BMI at each age

1) Cole TJ, Bellizzi MC, Flegal KM, Dietz WH. Establishing a standard definition for child overweight and obesity worldwide: international survey. *BMJ* 2000;320:1240-3.

2) World Health Organization. Obesity: Preventing and managing the global epidemic - Report of a WHO Consultation on Obesity (Geneva, 3-5 June 1997). Geneva, 1998

**Supporting information table S4: Unadjusted (model 1) and fully-adjusted (model 4) differences in mean grip strength at age 46y per 1 standard deviation increase in BMI at ages 10 to 46y and in BF% and WHR at age 46y among the sample who completed the grip strength assessment standing unsupported (N=6,890)**

|  |  | **Differences in mean grip strength (kg) (95% CI)** | | |
| --- | --- | --- | --- | --- |
|  | **Model** | **Males (N=3,388)** |  | **Females (N=3,502)** |
| BMI at age: |  |  |  |  |
| 10y | 1  4 | 1.37 (1.02,1.71)  1.03 (0.74,1.32) |  | 0.61 (0.41,0.80)  0.65 (0.47,0.84) |
|  |  |  |  |  |
| 16y | 1  4 | 1.07 (0.79,1.35) 1.33 (1.03,1.63) |  | 0.61 (0.40,0.81)  0.73 (0.53,0.92) |
|  |  |  |  |  |
| 30y | 1  4 | 1.36 (1.07,1.65) 1.37 (1.02,1.71) |  | 0.22 (0.03,0.41)  0.48 (0.30,0.67) |
|  |  |  |  |  |
| 46y | 1  4 | 1.03 (0.74,1.32) 1.07 (0.79,1.35) |  | 0.25 (0.06,0.44)  0.51 (0.33,0.70) |
|  |  |  |  |  |
| BF% at 46y | 1  4 | 0.39 (0.10,0.68)  0.40 (0.11,0.69) |  | 0.30 (0.12,0.49)  0.29 (0.11,0.47) |
|  |  |  |  |  |
| WHR at 46y | 1  4 | -0.04 (-0.33,0.26)  0.07 (-0.22,0.37) |  | 0.30 (0.12,0.49)  0.29 (0.11,0.47) |

Model 4 adjusted for height at 46y, father’s occupational class at birth and physical activity at age 10y, educational level attained, own occupational class and physical activity at age 46y

Note: results are combined from analyses run across 20 imputed datasets

**Supporting information table S5: Unadjusted (model 1) and fully-adjusted (model 4) differences in mean grip strength at age 46y per 1 standard deviation increase in BMI at ages 10 to 46y and in BF% and WHR at age 46y excluding those participants classified as severely hampered according to the European Statistics of Income and Living Conditions (EU-SILC) classification disability definition or with missing disability data (N=7.092)**

|  |  | **Differences in mean grip strength (kg) (95% CI)** | | |
| --- | --- | --- | --- | --- |
|  | **Model** | **Males (N=3,478)** |  | **Females (N=3,614)** |
| BMI at age: |  |  |  |  |
| 10y | 1  4 | 0.83 (0.53,1.12)  0.91 (0.61,1.20) |  | 0.66 (0.47,0.84)  0.72 (0.55,0.89) |
|  |  |  |  |  |
| 16y | 1  4 | 1.45 (1.10,1.79)  1.46 (1.12,1.80) |  | 0.70 (0.49,0.90)  0.80 (0.61,0.99) |
|  |  |  |  |  |
| 30y | 1  4 | 1.11 (0.83,1.40)  1.14 (0.86,1.42) |  | 0.32 (0.14,0.50)  0.58 (0.40,0.76) |
|  |  |  |  |  |
| 46y | 1  4 | 1.33 (1.04,1.62)  1.36 (1.08,1.64) |  | 0.35 (0.17,0.54)  0.60 (0.42,0.78) |
|  |  |  |  |  |
| BF% at 46y | 1  4 | 0.41 (0.12,0.70)  0.41 (0.13,0.69) |  | 0.43 (0.26,0.61)  0.41 (0.23,0.58) |
|  |  |  |  |  |
| WHR at 46y | 1  4 | -0.02 (-0.31,0.27)  0.05 (-0.24,0.34) |  | -0.03 (-0.21,0.15)  0.14 (-0.04,0.32) |

Model 4 adjusted for height at 46y, father’s occupational class at birth and physical activity at age 10y, educational level attained, own occupational class and physical activity at age 46y

Note: results are combined from analyses run across 20 imputed datasets

**Supporting information table S6: Unadjusted (model 1) and fully-adjusted (model 4) differences in mean grip strength at age 46y per 1 standard deviation increase in BMI at ages 10 to 46y and in BF% and WHR at age 46y with inclusion of those participants unable to complete the grip strength assessments for health reasons^*^ (N=7,617)**

|  |  | **Differences in mean grip strength (kg) (95% CI)** | | |
| --- | --- | --- | --- | --- |
|  | **Model** | **Males (N=3,691)** |  | **Females (N=3,926)** |
| BMI at age: |  |  |  |  |
| 10y | 1  4 | 0.75 (0.45,1.04)  0.83 (0.54,1.13) |  | 0.58 (0.39,0.77)  0.65 (0.47,0.82) |
|  |  |  |  |  |
| 16y | 1  4 | 1.33 (0.98,1.68)  1.38 (1.03,1.72) |  | 0.57 (0.37,0.77)  0.70 (0.51,0.89) |
|  |  |  |  |  |
| 30y | 1  4 | 1.03 (0.75,1.31)  1.06 (0.79,1.34) |  | 0.17 (-0.01,0.35)  0.46 (0.28,0.64) |
|  |  |  |  |  |
| 46y | 1  4 | 1.24 (0.95,1.52)  1.30 (1.02,1.58) |  | 0.15 (-0.03,0.33)  0.45 (0.28,0.63) |
|  |  |  |  |  |
| BF% at 46y | 1  4 | 0.36 (0.08,0.65)  0.40 (0.12,0.68) |  | 0.22 (0.05,0.40)  0.25 (0.08,0.43) |
|  |  |  |  |  |
| WHR at 46y | 1  4 | -0.14 (-0.43,0.15)  -0.01 (-0.30,0.28) |  | 0.15 (-0.03,0.33)  0.45 (0.28,0.63) |

Model 4 adjusted for height at 46y, father’s occupational class at birth and physical activity at age 10y, educational level attained, own occupational class and physical activity at age 46y

Note: results are combined from analyses run across 20 imputed datasets

* 70 participants unable to complete the grip strength tests for health reasons were included by allocating them grip strength values equivalent to the mean of the bottom sex-specific fifth

**Supporting information figure S1: Scatter plots and locally weighted regressions of BMI (kg/m^2^) vs grip strength (kg) for males at 16y and 46y**

Footnote: For brevity we present plots for BMI at ages 16 and 46y in males only as these models had the two largest quadratic terms (see supplementary table 2)
